# Remarks and Recommendations on the Professional Education of Dentists

**Published:** 1852-03

**Authors:** 


*Remarks and Recommendations on the Professional Education of Dentists.* By JOHN TRENOR, M. D., of New York. [From the New York Journal of Medicine.]

This pamphlet presents a very judicious and forcible argument in support of the position, "that a thorough knowledge of medicine and surgery is absolutely essential to enable the practitioner of dentistry clearly to understand and successfully to treat the cases which are constantly coming under his care." Dr. Trenor very decidedly coincides in the opinion lately expressed in this journal, by our correspondent, Dr. E. B. GARDETTE, that the *dental colleges*, brought into existence within a few years, are inefficient to remedy the deficiences which have existed in dental education; and he maintains that they can be supplied to advantage "only in a well organized medical school." These views, we believe, are entertained by a large proportion of the best educated practitioners of dentistry in this country; and we learn that the propriety of attaching a chair of dental surgery to some of our Faculties of Medicine, has been recently urged and seriously canvassed.

